# Quantifying the damage caused by fruit bats to backyard lychee trees in Mauritius and evaluating the benefits of protective netting

**DOI:** 10.1371/journal.pone.0220955

**Published:** 2019-08-15

**Authors:** Simon Tollington, Zainal Kareemun, Arlaine Augustin, Kunal Lallchand, Vikash Tatayah, Alexandra Zimmermann

**Affiliations:** 1 North of England Zoological Society, Chester Zoo, Chester, United Kingdom; 2 Durrell Institute of Conservation and Ecology, University of Kent, Canterbury, United Kingdom; 3 Mauritian Wildlife Foundation, Vacoas, Mauritius; 4 Wildlife Conservation Research Unit, Department of Zoology, Recanati-Kaplan Centre, University of Oxford, Tubney, Oxfordshire, United Kingdom; University of Lincoln, UNITED KINGDOM

## Abstract

The Mauritius fruit bat (*Pteropus niger*) has been the subject of repeated culling campaigns, apparently in response to pressure from the fruit-growing industry concerned over damage to commercially valuable orchard crops such as lychees. More than 31,000 fruit-bearing lychee trees also exist in private backyards, making this an issue pertinent to a wide cross-section of the Mauritian general public and not just those involved in commercial fruit production. The level of damage caused by bats to fruit crops is often debated and the low number of robust damage assessment studies hampers mitigation efforts. During the fruiting season of 2016/2017, we assessed the damage among backyard lychee trees attributable to fruit bats and other causes around Vacoas-Phoenix, Central Mauritius and evaluated the impact of using protective netting as a mitigation strategy. Fruit yield from panicles that were protected from depredation by nylon netting was approximately one third greater than that from unprotected panicles. We suspect that fruit bats were responsible for approximately 42% of the total damage but illustrate the difficulties in attributing damage to a single cause in such assessments. Although we demonstrate the value of protective netting, we recognize that barriers to implementation exist and that a more holistic approach that incorporates crop protection, forest restoration strategies and addresses negative public attitudes towards bats in general is required to ensure the persistence of this endemic species.

## Introduction

Fruit-bats (Pteropodidae) are recognised as important for the maintenance of healthy ecosystems and the provision of vital ecosystem services [1,2,3]. For example, they have been implicated in the proliferation of at least 289 plant species and at least 186 of these are known to provide extrinsic human benefits including timber, food and medicine [4,5]. They are however, threatened with habitat destruction and persecution throughout their distribution which spans tropical and subtropical Asia, Africa and Oceania [3]. Of 188 species recognised by the IUCN, 42% are considered of conservation concern (NT = 7%, VU = 20%, EN = 8%, CR = 4%, EX = 2%) and 47% least concern (11% are data deficient [6]). Approximately 50% of all pteropodid species are hunted for food [7] which contributes to their decline in many countries [8,9]. Where they threaten economically valuable fruit crops, fruit-bats are often shot or trapped in nets by fruit growers [10,11]. This persecution is perhaps compounded by widely-held negative attitudes among people toward bat species in general, associated with fear, disdain and disease [12,13,14,15].

Aziz et al., [3] provide an extensive review of the conflict between the fruit growing industry and pteropodid bats citing their persecution in a number of countries including Malaysia [16], Cyprus [17], and Australia [18], often as an apparently easy alternative to using deterrents or protective methods such as erecting nets. This conflict has resulted in legal culling in some countries, extending to eradication attempts in Israel, Australia and South Africa [19,3].

The Mauritius fruit bat or Mascarene’s flying fox (*Pteropus niger*) has recently been the subject of a culling campaign after mounting pressure from the public and the fruit-growing industry concerned at the loss of fruit. According to Government census, more than 31,000 lychee trees of fruit-bearing age existed in residential properties in Mauritius in 2011 [20]. It is therefore estimated that on average, 10% of all households have such a tree in the backyard, making this an issue for many residents as well as fruit growers with commercial interests [20]. Government-sanctioned culls instigated in 2015 and 2016 resulted in the removal of more than 38,000 individuals [21] and a more recent cull in 2018/2019 aimed to remove a further 20% (~13000) of the remaining population, despite evidence that this species performs a vital role in maintaining and regenerating native forest habitats and associated [22,23]. A recent Red List reassessment of this species reclassified it from ‘Vulnerable’ to ‘Endangered’ as a result of a 50% population decline between 2015 and 2017 largely attributed to the culls [24]. Pre-cull population estimates of this endemic fruit-bat are debated but range between 50,000 and 99,000 [25,26,24] with the number of mature individuals estimated between 16,000 and 33,000 [27]. A population assessment conducted after the cull in 2016 resulted in an estimate of ~62,000 individuals [28]. The culls have drawn widespread international criticism [25,29, 26,30,31,6] with opponents claiming that it increases the risk of extinction of an already vulnerable species [25,26,31], and that evidence from other countries including Australia suggests that culling is an ineffective method of reducing conflict that raises welfare and ethical issues [25,30]. Furthermore, there is no scientific evidence to suggest that culling of bats leads to a reduction in fruit damage [30]. There is however, little question that bats do indeed cause some damage to fruit crops in Mauritius but the level of that damage is often debated and other causes such as weather, invasive species and over-ripening are often overlooked [32].

Despite numerous records, reports and claims of the damage that Yinpterochiroptera cause to commercially valuable fruit crops globally [3], there are surprisingly few robust studies in the peer-reviewed literature that attempt to quantify actual damage. A recent study from Mauritius concluded that, within four orchards, bats were responsible for damaging approximately 25% of fruits on lychee and mango trees and that small trees were prone to less damage [32]. Similarly a separate study performed by the Food and Agricultural Research Extension Institute (FAREI) of the Government of Mauritius estimated the damage due to bats in lychee orchards at 10% [3].

The issue of bats damaging fruit is however, not limited to orchard owners or those involved in commercial production. To our knowledge there are no studies of damage to the 31,000 fruit trees located in private backyards in Mauritius that contribute substantially to this market. We aim to fill this knowledge gap by quantifying the level of damage to backyard lychee fruits caused by Mauritius fruit bats and the extent to which protective nets could ameliorate this damage.

## Methods

### Determining incidental fruit loss from trees

**Fig 1:**
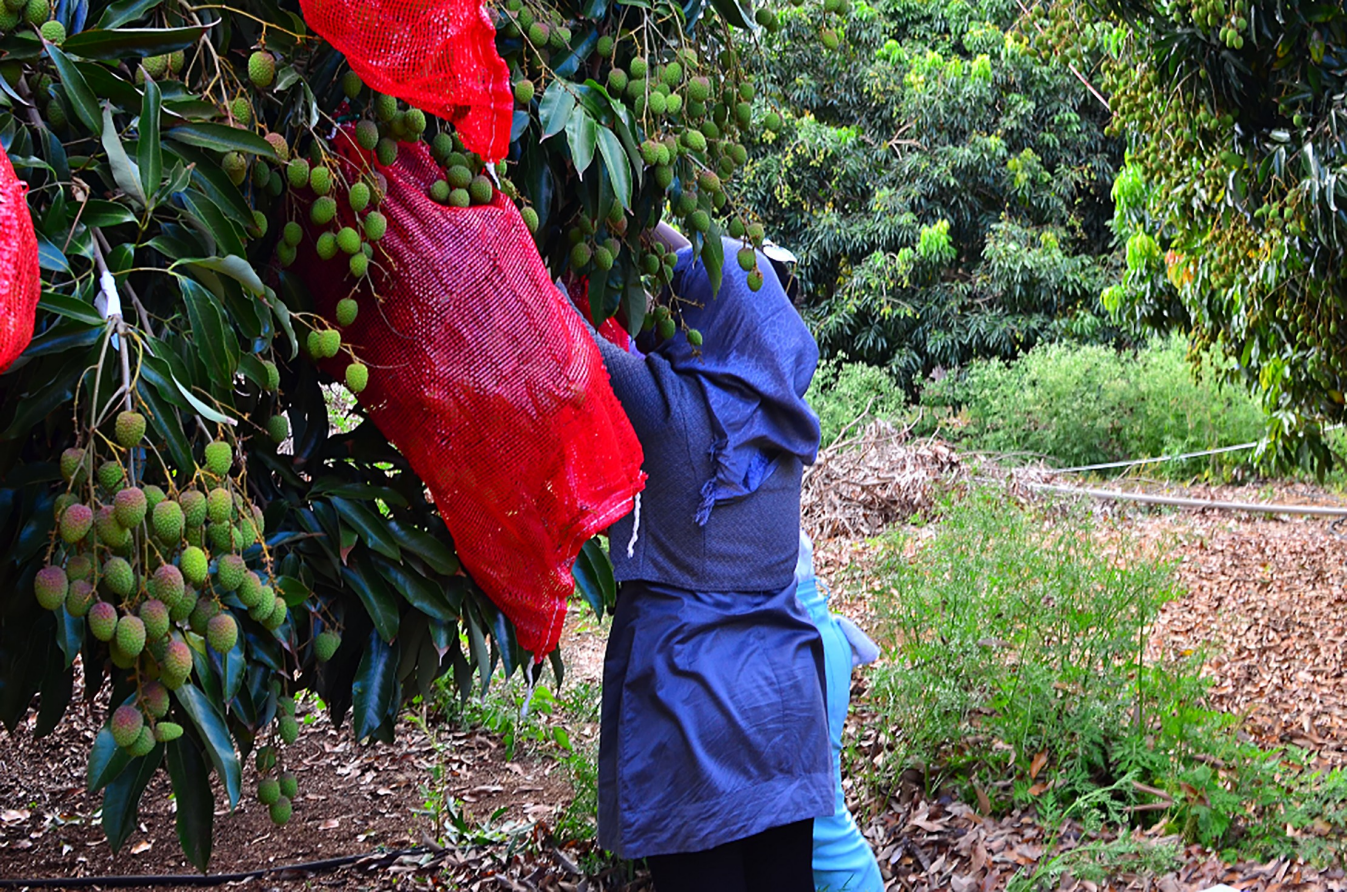
Image of the nylon netted bags used to protect lychee panicles.

To quantify damage caused by fruit bats to lychee fruit we adopted and modified the method described by Tillman et al., [33] that was originally developed to measure the extent of damage to fruit trees in Florida caused by birds. During the fruiting season (November–January) of 2016/17, we identified easily accessible, privately-owned backyard lychee trees in towns on the central plateau of Mauritius (-20.30, 57.50). Yield from these trees is rarely accurately known, they are often not managed and frequently as tall as the neighbouring houses. This densely populated area is situated to the north of the Black River Gorges National Park and therefore many of our trees were situated just a few kilometres of large fruit bat roosts. Trees were selected in the towns of Quatre Bornes, Beau Bassin-Rose Hill, and Vacoas-Phoenix in order to represent as wide an area as possible. After permission was sought from the land-owners to assess each tree, we identified 12 panicles that were representative of the fruit production of the whole tree and aimed to select panicles at different heights using small stepladders where necessary to access them; identifying them as being in the top, middle or bottom third of the tree. Selected panicles were then labelled with coloured flagging tape, attributed an individual identification code, and all damaged and underdeveloped fruits were removed. The number of lychees was then counted and recorded for each panicle and six of the panicles on all trees were then enclosed within a bag made of nylon net (sourced from a local vegetable market) as controls to exclude birds and bats (e.g. Fig 1). Thus, fruit that dropped into the net bags could be attributed to incidental loss (including here insect damage, natural drop and/or damage attributable to fungal infections, pathogens or nutrient deficiency). We returned to each tree at intervals of two or three days to repeat the counting exercise, and recorded the number of lychees that had fallen into the bags. This counting exercise continued until the owners of the trees harvested the fruit or installed their own protective netting. Our study did not directly involve endangered or protected species.

### Determining the cause of lost fruit

During each initial visit, we cleared all of the fallen fruit on the ground from underneath each tree. At all subsequent visits the fruits that had fallen from the tree were collected, counted and categorised. Each fallen fruit was assessed for damage and attributed one of the following criteria according to a guide published by the Department of Natural Resources and Environment of the Victoria State Government, Australia [34]:

‘*No signs of damage*’–was recorded when the fruit displayed no apparent external signs of depredation, or any other imperfections.

**Fig 2:**
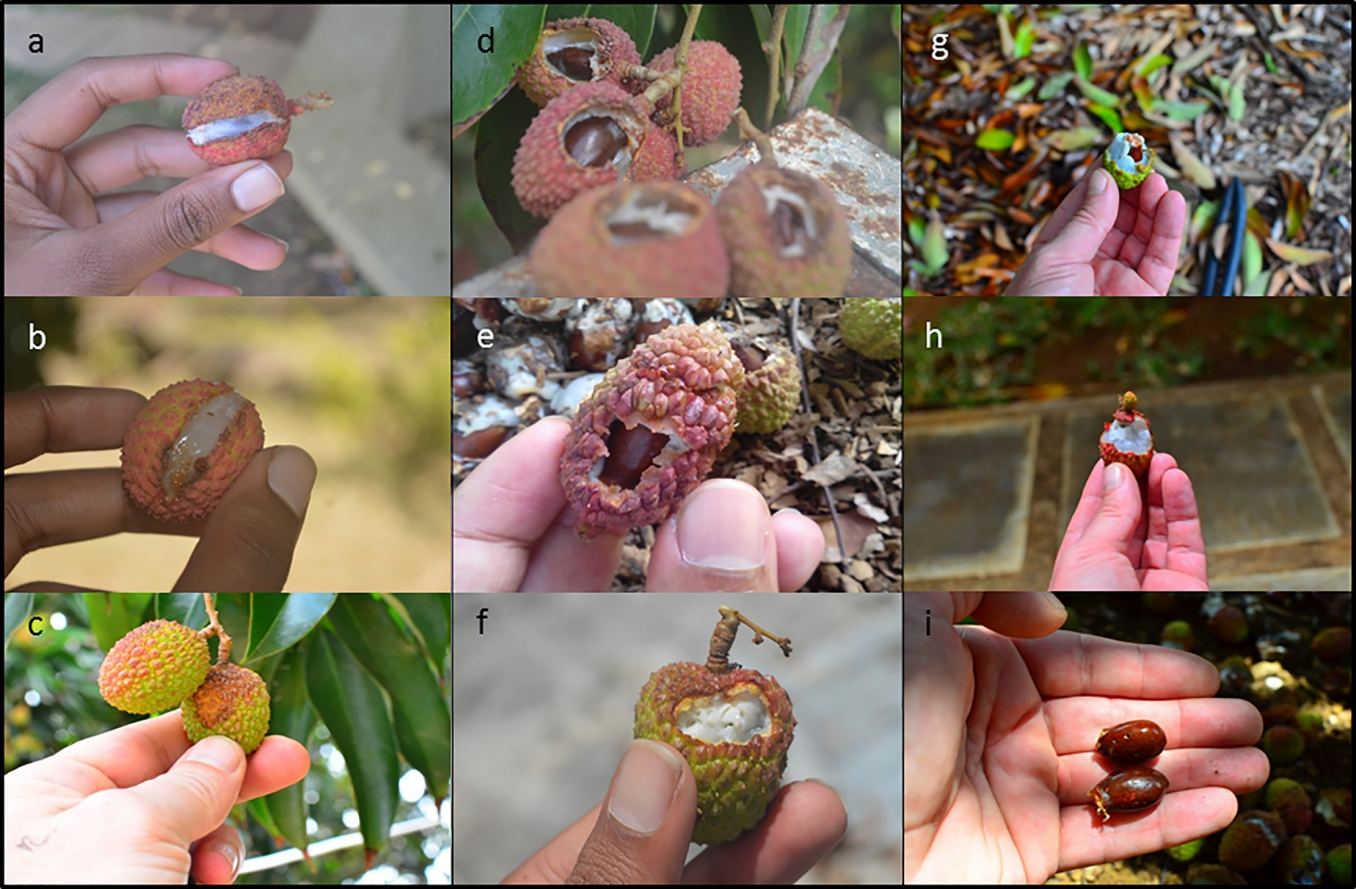
Images of lychee damage and classification. Natural split (a, b), unknown (c), bird damage (d, e and f), and bat damage (g, h and i).

‘S*plit’*,*—*(Fig 2A and 2B) was recorded when the fruit appeared to have split naturally leaving a neat linear opening with the flesh intact.

*‘Unknown damage*’–(Fig 2C) was recorded when the fruit had brown or rotten patches of skin (most likely caused by nutrient imbalance, insect damage or pathogen/fungal infection) or some other signs of damage not consistent with that caused by bats or birds as below.

‘*Damage by birds*’–(Fig 2D, 2E and 2F) was recorded when a neatly trimmed opening in the skin was discovered around the consumed fleshy interior.

‘*Damage by bats’*–(Fig 2G, 2H and 2I) was recorded when either a) an obvious bite-mark with jagged edges and chunk of fruit missing was apparent, or b) just the seed of a fruit was recovered.

All fallen fruit was cleared from the ground after counting and categorisation.

## Statistical analysis

To characterise fruit loss from lychee trees, we used Generalized Linear Mixed Models (GLMMs) to quantify the proportional loss of fruit attributable to either incidental damage or damage by birds or bats. The number of individual fruit counted per panicle was used as the response variable and three fixed factors were included as explanatory covariates: ‘Netted’ was a two-level factorial variable that described the treatment of netting a panicle or not; ‘Period’ was also a two-level factorial variable that differentiated between the initial and final fruit count; ‘Height’ was included as a three-level factorial variable that described the height of the panicle in relation to the tree as top, middle or bottom. We included as a random effect, ‘TreeID’ in order to account for the pseudoreplication of sampling multiple panicles per tree. The R package lme4 [35] was used to implement a GLMM to model the response variable with Gaussian errors since it was not zero-inflated and exploratory analyses [36,37] suggested that the residuals approximated a normal distribution. Our initial model included all fixed covariates in a full factorial design that incorporated all interaction terms. By subtracting the predicted average incidental fruit loss of netted panicles from the actual fruit loss of each non-control panicle we were able to estimate the proportion of fruit damaged by birds and bats that could be avoided with the use of nets.

Fruit that was collected from the ground was assessed in a similar way in an attempt to describe the proportion of lost fruits damaged by bats. The number of fruits collected was modelled in a GLM using a two-column vector to describe the proportion of fruits damaged for each tree [38]. A single, categorical predictor variable was included containing multiple levels that corresponded with our observational assessments of damage as previously described: *none*, *split*, *birds*, *bats*, or *unknown*. TreeID was initially included in this model as a fixed effect in order to account for the variation in fruit-drop between trees but this model was over-dispersed and was therefore fitted with a quasi-binomial error structure. We subsequently removed TreeID as a fixed covariate because model comparisons using F tests revealed that its inclusion was not important [38]. Our final model therefore, consisted of a GLM using a two-column response variable, a single multilevel fixed covariate and quasi-binomial error structure. We assessed goodness-of-fit for our models using R^2^ values from the R package piecewiseSEM to estimate the proportional variance explained by each of them [39].

## Results

A total of 20 lychee trees were assessed. Two of these were removed from the analyses due to extensive damage apparently caused by monkeys (*Macaca fascicularis*), no other trees were similarly affected. Several individual panicles were also removed from the analyses, where for example, whole branches had disappeared probably due to human disturbance. The final dataset therefore consisted of 18 trees and a total of 156 individual panicles, 81 of these panicles were not protected with a net bag and 75 were. Each panicle was monitored over a period of, on average, 18 days before the tree was harvested or a net was placed over it by the owner.

Our initial model (Table 1) revealed that enclosing panicles within net bags significantly reduced the amount of fruit lost. On average ~11(95%CI 7–15) fruits were lost per panicle between the initial and final counts (Table 2). The significant interaction effect of the model suggested that placing a netted bag over panicles saved almost 9.5 (95%CI 4–15) of those. Model fitted values (Table 2) revealed that the proportional fruit loss from panicles enclosed within netted bags was ~4%, whilst that lost from panicles that were not netted was ~37%. Subtracting one from the other revealed that, on average, and without protective netting, the loss of fruit through damage attributable to birds, bats and other factors preventable by netting, accounted for ~33% (95%CI 13–53) of potential yield.

**Table 1:** Output of a GLMM to identify predictors of fruit counts from panicles with 95% confidence intervals. Two fixed covariates of ‘Netted’ (True or False) and ‘Period’ (Initial or Final) were included. On average, significantly fewer fruits were counted on panicles at the end of the experiment, but panicles with nets suffered significantly reduced fruit loss compared to those without nets.

	Estimate	SE	df	t value	lower	upper
(Intercept)	29.41	2.47	29.51	11.89	24.50	34.29
NettedTRUE	3.76	1.93	290.51	1.95	-0.02	7.53
PeriodFINAL	-10.95	1.88	290.34	-5.82	-14.63	-7.27
NettedTRUE:PeriodFIINAL	9.44	2.73	290.34	3.46	4.11	14.77

Marginal R^2^ = 0.13, Conditional R^2^ = 0.43

**Table 2:** Model fitted values and 95% confidence intervals revealing average number of fruit per panicle according to the netting treatment between initial and final counts and proportional loss of fruit.

Netted	Period	Count	SE	lower	upper	% loss
FALSE	initial	29.41	2.47	24.55	34.28	
FALSE	final	18.46	2.47	13.60	23.33	37.23
TRUE	initial	33.17	2.51	28.23	38.10	
TRUE	final	31.65	2.51	26.72	36.59	4.56

**Fig 3:**
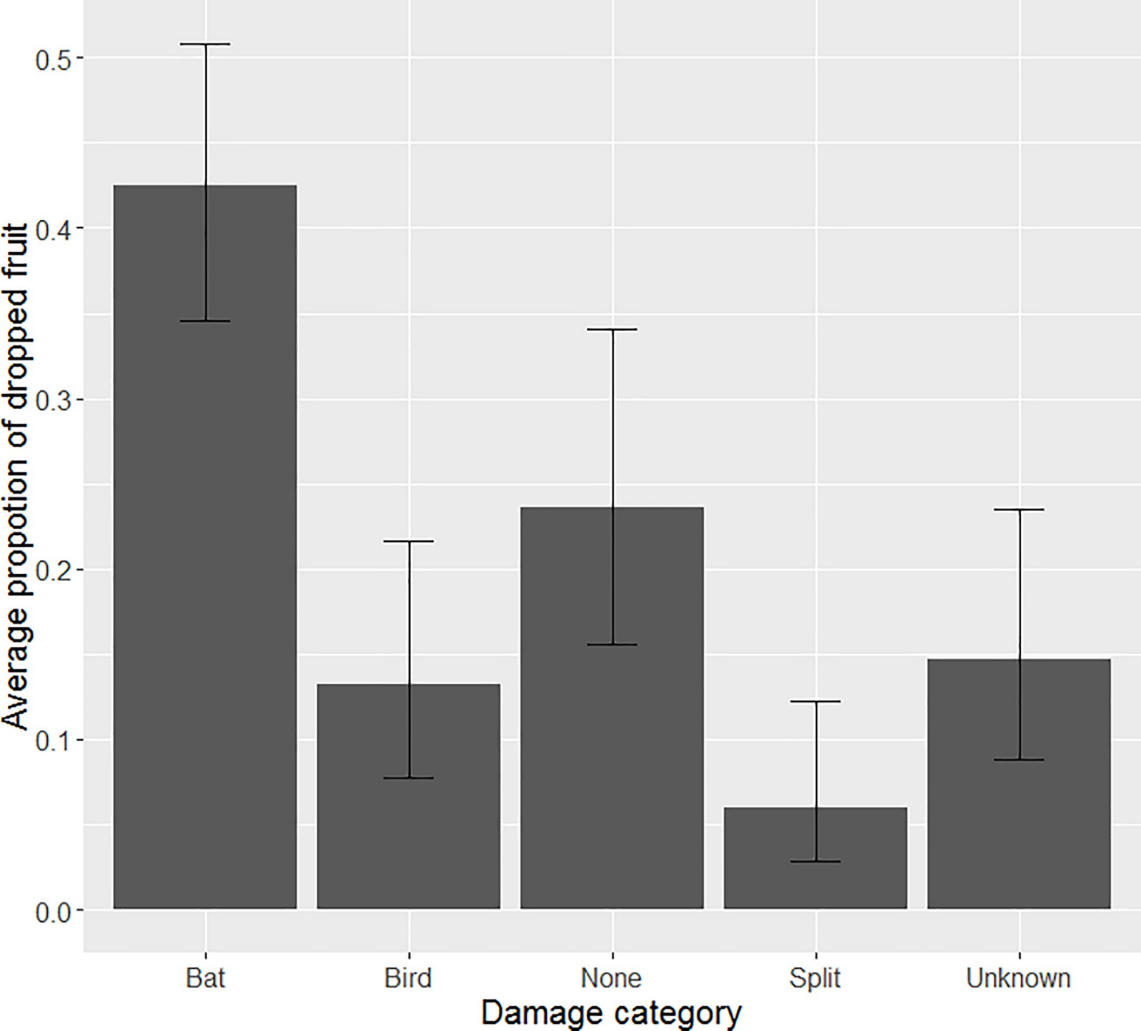
Causes of damage to dropped fruit. Average proportional damage (and 95% confidence intervals) to dropped fruit collected from underneath trees attributed to different causes.

We recovered, sorted and assessed a total of 4810 fruits from underneath lychee trees. Our second model revealed that, according to our damage assessment method, and on average across all the trees, bats were responsible for higher levels of damage than all other causes that we could identify (Table 3). Coefficients from the GLM were back-transformed to proportions [38] in order to reveal the relative damage and 95% confidence intervals of each category (Fig 3). According to these results we estimate that bats were responsible for, on average ~42% (CI 35–51) of the lychees collected from the ground underneath trees, ~24% (CI 16–34) of those collected showed no damage, ~15% (CI 9–24) revealed unknown damage, ~13% (CI 8–22) were damaged by birds and ~6% (CI 3–12) appeared to have split (Fig 3).

**Table 3:** GLM output characterising the cause of damage to fallen fruits. Upper and lower confidence intervals around the estimates do not cross zero revealing that damage attributable to bats is significantly higher than all other causes.

	Estimate	SE	t value	lower	upper
(Intercept) CauseBat	-0.30	0.17	-1.77	-0.64	0.03
CauseBird	-1.58	0.30	-5.19	-2.20	-1.00
CauseNone	-0.87	0.26	-3.31	-1.40	-0.36
CauseSplit	-2.45	0.40	-6.17	-3.29	-1.72
CauseUnknown	-1.46	0.30	-4.93	-2.05	-0.89

Marginal R^2^ = 0.45

## Discussion

### The value of netting and barriers to implementation

We have demonstrated that protective netting can be used as an effective method to reduce the depredation of lychees by fruit bats in backyards. Our results suggest that, on average, fruit yield from backyard lychee trees can be increased by approximately one third by correctly installing protective nets. This average value however, should be considered in the context of our study where household owners typically have one or two lychee trees in their backyard that can be decimated of fruit in a single evening. Indeed during our own study, out of 81 unprotected panicles at least 10 lost 100% of their fruit. The importance of protective netting should not therefore be understated and our findings agree with the assertions of many that full exclusion netting is perhaps the most effective method of mitigating damage to fruit trees from bats and birds [33,40,3]. Full canopy exclusion netting can completely eliminate damage by flying foxes if erected and maintained correctly [40,3]. However, the financial and practical implications of covering complete orchards or backyard trees with nets often provide barriers to implementation [3]. For example, many backyard lychee trees in Mauritius are sprawling and tall, making them very difficult to access and net effectively. Furthermore, the results from a questionnaire survey associated with this research (in preparation) reveal that many backyard growers do not maintain their trees for financial benefit. We identified 110 backyard growers and 67% of these did not sell their fruit, preferring instead to keep the produce for their own consumption and to share it among friends and family (in preparation). This perhaps goes some way to explain why a large proportion of backyard growers (86% of 97 who answered) do not employ netting to protect their trees. It is possible that the perceived benefit of erecting nets does not outweigh the financial and practical costs of doing so.

### The implementation of netting subsidies

The Government of Mauritius has made a significant effort to encourage orchard owners and backyard growers to increase their use of netting by providing a subsidy scheme for the purchase of nets since 2009. Under the scheme, growers can buy a certain number of nets at 75% discount. In 2015 and 2016 the Government invested Rs55m (>$1.5mUSD) to support more than 8000 applications for protective netting, demonstrating its commitment to finding alternative, non-lethal methods of conflict mitigation [21,28]. The subsidy system implemented by the Government of Mauritius is a laudable and generous attempt at increasing the use of nets in order to mitigate damage by non-lethal means, however the uptake of this financial incentive appears to have been low among growers with many claiming to be unaware of the subsidy scheme. However, increasing awareness of the available financial aid is unlikely to remove all barriers to implementation, nets still cost money, are difficult to erect in backyards and are not perceived by most people as a valuable financial investment.

### Tree management

Maintaining fruit trees with regular pruning is often seen as an important management action among orchard owners in Australia that facilitates the cost-effective implementation of full exclusion netting with permanent frames and simultaneously increases yield by isolating tree canopies (I. Groves pers. comm. November 2017). Many orchard owners in Mauritius however, claim that they cannot afford to invest the initial capital required to properly install permanent frames, or to lose future yield by pruning fruit-bearing trees to make them easier to net. To date there is no evidence from Mauritius concerning the economic implications of pruning trees in order to facilitate easier netting and this is therefore a strategy that requires further assessment.

### Attributing cause of damage

Ours is the first study to document the loss of fruit from trees that are not part of commercial orchards and therefore demonstrates that this issue is not unique to orchard owners but also affects a substantial proportion of the general public. Our assessment of fruit that had fallen from trees revealed that bats were responsible for a substantial proportion of lost fruit from backyards, significantly more than any other identifiable cause. However, we recognise that our method of identifying a single cause of damage for each fruit must be considered alongside known limitations. The certainty of attributing damage of lychees to different depredating taxa and parasites/pathogens cannot be guaranteed and remains a challenge for these types of assessment studies. For example, damage that appears to have been caused by birds could actually be damage caused by splitting that has then allowed birds to access the fruit, thus the original damage in this case would not be bird-related. Imperfections in lychees, many of which make them unsuitable for sale or consumption, can be caused by a variety of factors including over ripening, natural split, nutrient imbalance, fungal infections, excessive heat, insect damage, parasites and pathogens [41]. These imperfections can allow birds and other vertebrates easy access to the flesh and a proportion of the fruit consumed by bats will therefore also have already been damaged before being depredated. Thus, identifying a single culprit associated with any type of damage and estimating loss of yield accordingly is largely subjective and can rarely be confirmed with certainty. Furthermore, fruits that fall to the ground with no apparent damage may be an indirect effect of bats or birds landing and moving around the tree, or indeed they may have fallen due to high winds or natural drop. Additionally, our study cannot quantify the number of fruits that may have been removed from the tree for consumption elsewhere.

Rats are also suspected to be responsible for significant damage to lychee fruits (despite a lack of evidence) but it is very difficult to distinguish rat damage from bat damage; it is suspected that rats gnaw on the exposed seed of the lychee leaving recognisable, chisel-like patterns, but it is possible for example, that the seed is first exposed by bats (or birds) and then secondarily depredated by rats after it falls to the ground. Additionally, given that rats are known hoarders [42,43] we suspected that there would be very little evidence of rat consumption and so did not include rat damage in our assessment.

In order to improve the accuracy of damage attribution in this system, an experimental approach to excluding certain taxa could be applied. For example, Maas et al., [44] successfully implemented a series of exclusion experiments to study the effects of birds and bats on insect herbivore abundance, a similar initiative could be implemented in Mauritius to more accurately characterise the loss in yield under a range of scenarios that exclude certain taxa by erecting nets with a variety of mesh sizes. Alternatively, observational monitoring combined with damage assessment may provide evidence to separate bird damage (day-time observations) and bat damage (night-time) observations.

### What is the cost of damage?

It is of no real surprise that bats cause a considerable amount of damage to fruit crops in Mauritius as this has long been the general consensus among fruit growers and the general public even if robust evidence from multiple locations was lacking. We recovered 4810 fruits from underneath backyard lychee trees and according to our estimate approximately 4.5% (216) of this could be attributed to natural drop. Therefore 4594 fruits were lost from 18 trees that could have been avoided with the use of full exclusion netting. In the 2011 Housing and Population Census of Mauritius a total of 31068 residential lychee trees were recorded (Statistics Mauritius 2011). Crude extrapolation suggests that if 255 fruits are lost per tree (4594/18) across Mauritius, then the potential increase in yield under a scenario of full exclusion netting would be 31068*255 = 7,922,340 individual fruits. Assuming a market value of Rs2 per fruit this equates to Rs15.8m. Again, using crude extrapolation and assuming that our attribution of damage is accurate, bats could be responsible for (0.42*15.8) Rs6.6m worth of damage to backyard lychees alone. This is the equivalent of ~15% of the annual export value of lychees in Mauritius; estimated by the Food and Agricultural Research Extension Institute at Rs45-50 million (FAREI pers. comm. 2017)

### Adopting a holistic approach to damage reduction

Although our evidence supports others’ recommendations [32] that netting could prevent considerable losses of fruit, any damage mitigation strategies need to be incorporated into a holistic approach that recognises the need for improved protection and management of fruit trees, alongside wider forest regeneration, ecological restoration and cultural attitudes. Erecting nets can only be considered a technical ‘sticking plaster’ that does not address the underlying ecological predictors of fruit depredation by bats and may in fact simply displace the issue from one backyard or orchard that is netted to another that is not protected. Furthermore, assessing fruit damage attributable to bats and designing practical and effective mitigation techniques, although important, does not address the underlying negative perceptions of bats by the general public, which undoubtedly contributes support to the culling campaigns.

Significantly, Mauritius is an example of many oceanic islands that have been devastated by multiple extinctions and the introduction of a suite of non-native species resulting in increased competition for natural resources and a highly denuded forest ecosystem [45,46]. Less than 2% of Mauritius’ native flora remains intact [47] and large areas of remaining vegetation are heavily invaded with strawberry guava (*Psidium cattleyanum*). Crab-eating macaques (*Macaca fascicularis*), red-whiskered bulbuls (*Pycnonotus jocosus*) and rose-ringed parakeets (*Psittacula krameri*) are just three examples of the worst vertebrate invaders and macaques have been demonstrated to directly compete with fruit bats [48].

Lychees are only available during two months of the year so it is clear that fruit bats are able to persist on alternative sources of food (perhaps even non-native species) outside of this period. The Government of Mauritius is making progress with a forest restoration scheme and recent evidence suggests that in areas where strawberry guava has been removed, foraging activity of bats increases [48] demonstrating the value of this difficult task and illustrating the need for a holistic approach to sustainable and non-lethal solutions to this issue. Future research should focus on the seasonal foraging behaviour of fruit bats and availability of natural food in Mauritius in order to understand their dietary preferences, the effects of forest restoration and the role that fruit bats play in this. Importantly, the challenge of how to reduce the negative image of fruit bats that is widely held among the general public is yet to be resolved.
